# Tmax Determined Using a Bayesian Estimation Deconvolution Algorithm Applied to Bolus Tracking Perfusion Imaging: A Digital Phantom Validation Study

**DOI:** 10.2463/mrms.mp.2015-0167

**Published:** 2016-03-21

**Authors:** Ikuko Uwano, Makoto Sasaki, Kohsuke Kudo, Timothé Boutelier, Hiroyuki Kameda, Futoshi Mori, Fumio Yamashita

**Affiliations:** 1 Division of Ultrahigh Field MRI, Institute for Biomedical Sciences, Iwate Medical University, 2-1-1 Nishitokuta, Yahaba, Iwate 028-3694, Japan; 2 Department of Diagnostic and Interventional Radiology, Hokkaido University Hospital; 3 Department of Research & Innovation, Olea Medical

**Keywords:** perfusion imaging, Tmax, tracer arrival delay, mean transit time, Bayesian estimation

## Abstract

**Purpose:**

The Bayesian estimation algorithm improves the precision of bolus tracking perfusion imaging. However, this algorithm cannot directly calculate Tmax, the time scale widely used to identify ischemic penumbra, because Tmax is a non-physiological, artificial index that reflects the tracer arrival delay (TD) and other parameters. We calculated Tmax from the TD and mean transit time (MTT) obtained by the Bayesian algorithm and determined its accuracy in comparison with Tmax obtained by singular value decomposition (SVD) algorithms.

**Methods:**

The TD and MTT maps were generated by the Bayesian algorithm applied to digital phantoms with time-concentration curves that reflected a range of values for various perfusion metrics using a global arterial input function. Tmax was calculated from the TD and MTT using constants obtained by a linear least-squares fit to Tmax obtained from the two SVD algorithms that showed the best benchmarks in a previous study. Correlations between the Tmax values obtained by the Bayesian and SVD methods were examined.

**Results:**

The Bayesian algorithm yielded accurate TD and MTT values relative to the true values of the digital phantom. Tmax calculated from the TD and MTT values with the least-squares fit constants showed excellent correlation (Pearson’s correlation coefficient = 0.99) and agreement (intraclass correlation coefficient = 0.99) with Tmax obtained from SVD algorithms.

**Conclusions:**

Quantitative analyses of Tmax values calculated from Bayesian-estimation algorithm-derived TD and MTT from a digital phantom correlated and agreed well with Tmax values determined using SVD algorithms.

## Introduction

Various metrics generated by bolus tracking perfusion imaging such as magnetic resonance (MR) perfusion-weighted imaging (PWI) or perfusion computed tomography (PCT) have been used to evaluate areas of the ischemic penumbra in acute stroke patients, although these techniques are considered non-mandatory for guiding reperfusion therapies mainly because being time-consuming.^1,2^ Among the metrics, Tmax has been reported to accurately determine the penumbral area, given an appropriate cut-off value,^3,4^ and is currently used to quantitatively determine the penumbral area in clinical trials.^1,2,5^ Tmax is defined as the time to the peak of the tissue residue function, *R(t)*, obtained by singular value decomposition (SVD) deconvolution algorithms.^6^ Tmax theoretically depends on tracer arrival delay (TD), tracer dispersion, and mean transit time (MTT) in brain tissues.^6^ In practice, however, Tmax depends only on TD and MTT, because dispersion modulates MTT by the global arterial input function (AIF).^7^ A recently introduced deconvolution algorithm using Bayesian estimation overcomes known limitations of the SVD algorithms such as non-physiological oscillations of estimated *R*(*t*), vulnerability to image noise, and inherent inaccuracies in MTT and cerebral blood flow (CBF) values.^8^ The Bayesian method can directly estimate *R*(*t*) by applying Bayesian probability theory to the intravascular tracer model and calculate other perfusion metrics by reconvoluting the obtained *R*(*t*). Therefore, this technique can robustly estimate individual hemodynamic parameters, including CBF, MTT, and TD, without delay sensitivity.^8–10^ However, the Bayesian method cannot calculate Tmax directly, as it is a nonphysiological index of the distorted *R*(*t*) profile obtained by SVD algorithms, which reflects combined information of the TD and MTT.^6^ Hence, in this study, we attempted to establish a computational method to generate Tmax from the Bayesian method derived TD and MTT using correlations with Tmax values obtained by SVD algorithms.

## Materials and Methods

### Digital phantom

**Fig 1.**
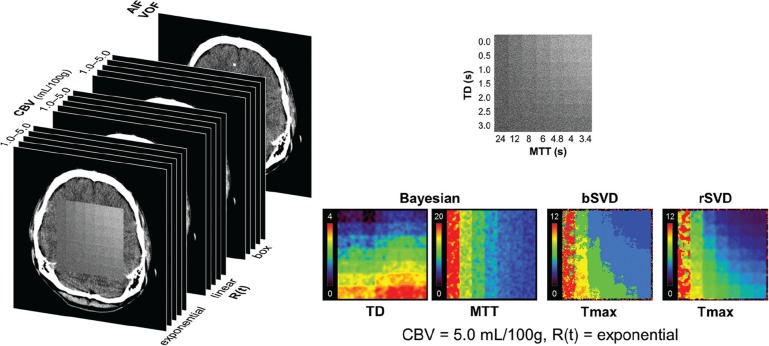
Data structure of the digital phantom and perfusion maps generated by different algorithms. The digital phantom consists of slices in which quadratic tiles of different tracer arrival delay (TD) and mean transit time (MTT) values are embedded in vertical and horizontal axes, respectively. Cerebral blood volume (CBV) and residue function, *R*(*t*), differ across the slices. Global arterial input function (AIF) and venous output function (VOF) are embedded in another slice. Color maps of TD and MTT are generated by the Bayesian algorithm, while color maps of Tmax are generated by block-circulant singular value decomposition (bSVD) and reformulated singular value decomposition (rSVD) algorithms. The TD and MTT maps by the Bayesian method appear comparable to the true values. Only slices with CBV of 5.0 mL/100 g and exponential *R*(*t*) are shown.

We used a digital phantom introduced in a previous study^11^ (available here: http://amrc.iwatemed.ac.jp/ibms-en/projects/cmri/download.html). This Digital Imaging and Communication in Medicine (DICOM) format phantom includes timedensity curves of brain tissue embedded in 7 × 7 quadratic tiles, as well as the global AIF and venous output function (VOF) of real brain computed tomography (CT) images at 16 slice locations taken every 2 s over a 60-s duration (Fig. 1). The time-density curves reflected the following variable parameter sequences: seven tracer arrival TD values (0.0 s, 0.5 s, 1.0 s, 1.5 s, 2.0 s, 2.5 s, and 3.0 s), seven MTT values (3.4 s, 4.0 s, 4.8 s, 6.0 s, 8.0 s, 12.0 s, and 24.0 s), five cerebral blood volume (CBV) values (1.0 mL/100 g, 2.0 mL/100 g, 3.0 mL/100 g, 4.0 mL/100 g, and 5.0 mL/100 g), and three kinds of *R*(*t*) (exponential, linear, and box-shaped). These parameter sequences were accompanied with Gaussian noise typical of PCT data scanned with 80 kVp at 200 mAs. In this phantom, the dispersion was incorporated into the MTT values using the global AIF.

### Data analyses

The digital phantom was post-processed using three software packages, each with different algorithms, as follows: (1) Olea Sphere (Olea Medical, La Ciotat, France) with the Bayesian estimation algorithm,^8^ (2) Perfusion Mismatch Analyzer (PMA) (Acute Stroke Imaging Standardization Group Japan, http://asist.umin.jp/index-e.htm) with the block-circulant SVD (bSVD) algorithm,^12^ and 3) CBP Study Ph8 (Toshiba Medical Systems, Tokyo) with the reformulated SVD (rSVD).^13^ The last two software packages with their associated algorithms provided the best benchmarks in a previous digital phantom validation study.^11^ The TD and MTT maps were generated by the Bayesian algorithm, while Tmax maps were generated by the bSVD and rSVD algorithms (Fig. 1). These maps were exported in DICOM format and then loaded in the PMA software for further analyses of multiple square regions of interest (ROIs).

Tmax values determined from Bayesian algorithm metrics, Tmax(Bayesian), were calculated by the following equation, which was simplified from a previous study:^6^

(1) Tmax (Bayesian)=TD (Bayesian)+p×MTT (Bayesian)+q,

where *p* and *q* are best-fit parameters determined for each SVD algorithm and TD(Bayesian) and MTT(Bayesian) are the TD and MTT values, respectively, obtained by the Bayesian method. The *p* and *q* values specific to each SVD algorithm were found by a linear least square method that minimized differences between Tmax(Bayesian) and the Tmax values of the SVD algorithms, Tmax(bSVD), and Tmax(rSVD). Pearson’s correlation coefficient (*r*) was calculated to evaluate correlations between Tmax(Bayesian) and Tmax(bSVD) or Tmax(rSVD) values. In addition, the slopes and intercepts of the linear regression lines, as well as intraclass correlation coefficients (ICCs), were also calculated to evaluate agreement between the Tmax values. Correlations and agreement of TD and MTT values obtained by the Bayesian method with ground truth values of the digital phantom were also evaluated using *r* and ICC. These analyses were performed using SPSS Ver. 19 (IBM, Chicago, IL, USA).

## Results

**Fig 2.**
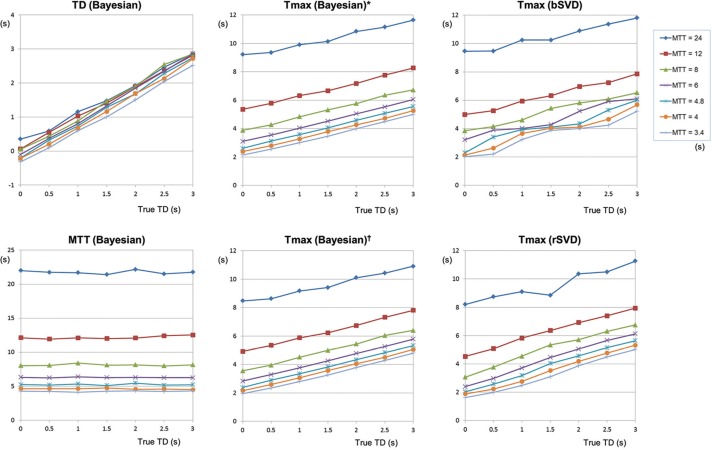
The TD, MTT, and Tmax generated by the Bayesian algorithm and Tmax generated by the SVD algorithms. The Bayesian algorithm generated TD and MTT values correlate well with true values. Tmax values calculated from the TD and MTT agree well with those generated by bSVD and rSVD. *p/q = 0.361/0.927; †*p/q* = 0.331/ 0.851. bSVD, block-circulant singular value decomposition; MTT, mean transit time; rSVD, reformulated SVD; TD, tracer arrival delay.

The TD, MTT, and Tmax maps were generated as described in the “Methods” section from the digital phantom (Fig. 1). The TD and MTT values obtained by the Bayesian algorithm showed excellent correlations (*r* = 0.98 and 1.00, respectively) and agreement (regression lines, y = 0.95x − 0.10 and y = 0.86x + 1.26, respectively; ICC = 0.97 and 0.99, respectively) with the true values of the digital phantom (Fig. 2).

The linear least squares best-fit values of *p* and *q* of Eq. (1) were 0.361 and 0.927, respectively, when fit to Tmax(bSVD), and 0.331 and 0.851, respectively, when fit to Tmax(rSVD) (Table 1).

**Fig 3.**
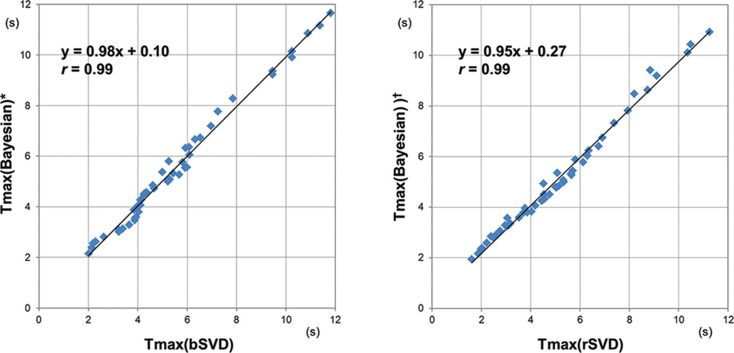
Linear regression analyses of Tmax(Bayesian) with Tmax(bSVD or rSVD). Tmax values calculated from the tracer arrival delay and mean transit time values obtained by the Bayesian algorithm show excellent correlation and agreement with those by the bSVD and rSVD algorithms. *p/q = 0.361/0.927; †*p/q* = 0.331/0.851. bSVD, block-circulant singular value decomposition; rSVD, reformulated SVD.

Tmax(Bayesian) calculated with the each set of *p* and *q* values had nearly identical dependencies on the true TD and MTT values as did the corresponding Tmax(bSVD) or Tmax(rSVD) (Fig. 2). Linear regression analyses showed that the Tmax(Bayesian) values resulted in excellent correlations with the Tmax(bSVD) and Tmax(rSVD) values (*r* = 0.99, for both) (Table 1, Fig. 3). In addition, the slope and intercept of the regression lines of Tmax(Bayesian) were 0.93 and 0.10, respectively, for Tmax(bSVD) as well as 0.95 and 0.27, respectively, for Tmax(rSVD) (Table 1, Fig. 3). Further, ICCs between Tmax(Bayesian) and Tmax(bSVD) or Tmax(rSVD) values were both 0.99 (Table 1). These results indicate excellent, nearly ideal agreements of Tmax(Bayesian) calculated from TD(Bayesian) and MTT(Bayesian) with Tmax(bSVD) or Tmax(rSVD), when using optimal *p* and *q* values that are specific to the SVD algorithms.

## Discussion

Tmax has been used to determine ischemic penumbral regions that were potential targets of reperfusion therapies in recent major clinical trials.^5,14–16^ Quantitative automated detection of hypoperfused areas with prolonged Tmax over certain cut-off values, e.g., 6 s, has been commonly adopted to determine perfusion-diffusion mismatch areas reflecting ischemic penumbra.^17^ Although Tmax is a wellestablished and reliable metric, it is also non-physiological and artificial, and includes biological and algorithmic information. A recent study showed that Tmax depends mainly on TD, dispersion, and, to a lesser extent, MTT, and can be expressed as a complex function of these parameters.^6^ However, in this study, we used a simple equation to calculate accurate Tmax values from only TD and MTT values obtained using a Bayesian estimation algorithm. Theoretically, under the global AIF, MTT values include tracer dispersion information,^7^ indicating that precise MTT measurements are required to calculate Tmax. A recent phantom study revealed that the Bayesian estimation method generated more accurate MTT values than did SVD methods.^9^ In addition, as demonstrated in this study, the Bayesian method can generate accurate TD values, which are largely inaccessible by other deconvolution or non-deconvolution algorithms. Therefore, the Bayesian method should be able to indirectly but readily generate Tmax comparable to those generated by SVD methods.

One of the advantages of the Bayesian method against the SVD methods is the capability to simulate Tmax values of other various algorithms, which can help compare the results with those in previous studies or other institutions. One inherent issue with a Tmax calculated by SVD is its dependence on the specific deconvolution algorithm.^6,11^ When calculating Tmax from TD and MTT obtained by the Bayesian algorithm according to Eq. (1), the constants *p* and *q* should be optimized for the individual SVD algorithms. Least squares fits to the Tmax of bSVD and rSVD returned different sets of optimizing *p* and *q*. Various software programs using different types of the SVD family, such as standard SVD (sSVD), bSVD, and oscillation-index regularized SVD (oSVD), have been used to obtain Tmax.^11^ In order to reproduce these SVD-derived Tmax values by the Bayesian method, the digital phantom we used could provide constants *p* and *q* specific to each algorithm. Other advantages of the Bayesian method against SVD algorithms include robustness to the noise, truncation, and oscillation,^8^ which can improve precision of the Tmax, although postprocessing time cannot be shortened.

There are several limitations in this study. First, the phantom we used included limited perfusion information. Tracer dispersion was inexplicit, but instead incorporated into the MTT data by the global AIF. The shape of *R*(*t*) is unknown in normal and hypoperfused brain tissues; hence, the shape used may have been non-realistic. We did not investigate the dependence of our results and analyses on the signal-to-noise ratio (SNR), which can affect perfusion analysis accuracy, although the PCT phantom we used had a low SNR. We did not perform a similar investigation using the PWI phantom because the SNR was higher than that of the PCT phantom and one of the PWI software programs showing the best benchmark was unavailable in this study; however, we assume that the method we introduced can readily regenerate Tmax on PWI by the Bayesian algorithm. We could not demonstrate comparisons of Tmax images obtained by the Bayesian and SVD methods in phantoms or clinical cases because the software was still unavailable to generate the Bayesian Tmax images, which will be overcome by modifying the program in the near future. We performed no comparisons between Tmax and other physiological parameters in acute stroke patients, because this issue is beyond the scope of this study. Thus, potential advantages of Tmax against other parameters obtained by the Bayesian method remain unclear. Further studies using MR and CT data from patients with acute stroke and revised software program will allow us to determine whether indirect estimation of Tmax from TD and MTT by the Bayesian method is accurate and can readily determine penumbral areas, relative to major SVD methods or major physiological parameters.

## Conclusion

Quantitative analyses of a digital phantom with a global AIF revealed that the Bayesian estimation algorithm readily calculated Tmax from TD and MTT. The Bayesian method Tmax values were nearly identical to the bSVD and rSVD algorithm Tmax values when calculated using individually optimized constants for each algorithm.

## Figures and Tables

**Table 1.** Correlation and agreement of Tmax obtained by Bayesian and SVD methods

	Tmax (Bayesian)						

	*p*	*q*	*r*	Slope	Intercept	ICC
Tmax(bSVD)	0.361	0.927	0.994	0.983	0.096	0.994
Tmax(rSVD)	0.331	0.851	0.994	0.947	0.273	0.993

bSVD, block-circulant singular value decomposition; ICC, intraclass correlation coefficient; *p, q*, constants relating Tmax to tracer arrival delay and mean transit time in Bayesian method (Eq. 1); *r*, Pearson’s correlation coefficient; rSVD, reformulated singular value decomposition; Tmax, time-to-maximum of tissue residue function.
